# Multiplexing fluorogenic esterase-based viability assay with luciferase assays

**DOI:** 10.1016/j.mex.2019.09.008

**Published:** 2019-09-12

**Authors:** Kenji Ohgane, Hiromasa Yoshioka, Yuichi Hashimoto

**Affiliations:** Institute for Quantitative Biosciences, The University of Tokyo, 1-1-1 Yayoi, Bunkyo-ku, Tokyo 113-0032, Japan

**Keywords:** CytoRed-luciferase multiplex assay, Multiplex assay, Luciferase, CytoRed, Esterase, Fluorogenic substrate, Viability

## Abstract

Luciferase-based reporter assays are one of the most common cell-based screening formats for drug discovery, and simultaneous evaluation of the cytotoxic effect of test compounds is of great value in reducing false-positives. Here we share a multiplex assay protocol that allows sequential measurement of cell viability (cell number) and luciferase activity of the same sample in a multi-well-plate format. The viability assay employs a fluorogenic esterase substrate, CytoRed.

• This protocol allows sequential measurement of endogenous esterase activity (as a surrogate for cell number) and then luciferase activity in a single sample.
• The protocol eliminates the need for parallel viability assay or protein assay using separate aliquots of the lysate.
• This protocol is especially useful for assays with cells stably expressing a luciferase construct, for which co-transfection of another reporter gene is not a viable option.

## Graphical abstract

**Figure:**
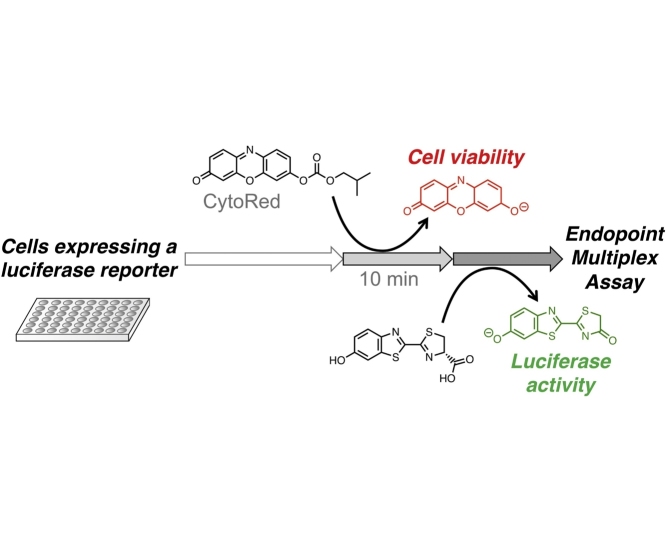

**Specification Table**

**Table:** 

Subject Area:	*Biochemistry, Genetics and Molecular Biology*
More specific subject area:	*Cell biology / medicinal chemistry / screening technology*
Method name:	*CytoRed-luciferase multiplex assay*
Name and reference of original method:	***Viability assay with CytoRed:****M. Ishiyama, H. Furusawa, M. Shiga, F. Ohseto, K. Sasamoto, A Resorufin Derivative as a Fluorogenic Indicator for Cell Viability, Anal Sci 15 (1999) 1025–1028.* ***Multiplex assay of luciferase and resazurin-based viability assay:****J. Herbst, M. Anthony, J. Stewart, D. Connors, T. Chen, M. Banks, E.W. Petrillo, M. Agler, Multiplexing a high-throughput liability assay to leverage efficiencies, Assay and Drug Development Technologies 7 (2009) 294–303.* ***Live-cell multiplexed reporter gene assays monitoring the cell viability and the compound kinetics on luciferase activity****: M.-C. Didiot, S. Serafini, M.J. Pfeifer, J.K. Martin, J. Frederick, C.N. Parker, Multiplexed Reporter Gene Assays: Monitoring the Cell Viability and the Compound Kinetics on Luciferase Activity, J. Biomol. Screen 16 (2011) 786–793.*
Resource availability:	*CytoRed reagent can be easily synthesized, or is commercially available from multiple vendors.*

## Method details

Cell-based reporter assays are widely used in drug discovery programs, and luciferase is one of the most widely used reporter enzymes due to its high sensitivity and wide dynamic range. In cell-based high-throughput screening, cytotoxic compounds are a major source of false positives, so multiplex measurements of cell viability and luciferase activity can improve the accuracy of hit selection and reduce the time and cost of secondary, confirmatory assays.

In luciferase-based reporter assays involving transient transfection, normalization of the luciferase activity is essential to account for well-to-well variations of transfection efficiency and cell number. For this purpose, an additional reporter plasmid encoding an orthogonal enzyme, such as beta-galactosidase [1] or *Renilla* luciferase [2,3] is co-transfected, and its enzymatic activity is used for normalization. In some cases, however, monitoring processes of interest requires the use of a stable cell line expressing the luciferase-based reporter. For example, assays designed to monitor the stability of a protein of interest by fusing a luciferase to it often require the establishment of stable cell lines to obtain a reproducible response [4]. Then, the requirement of a stable cell line makes the co-transfecting strategy impractical. For normalization purposes, cell viability assays are sometimes performed in parallel, or total protein concentration is occasionally determined from aliquots of the lysate [5]. However, such protocols require additional resources or multiple steps for protein assay, and are not practically suitable for high-throughput assays on 96- or 384-well plates.

Several protocols, which combine resazurin-based cell viability assay or protease-based viability assay with luciferase assay, have been developed to enable multiplex determination of cell viability (number) and luciferase activity [[6], [7], [8]]. Although these protocols work well for assays monitoring relatively slow processes, such as changes of mRNA stability or transcriptional activity, they are not suitable for monitoring faster processes, including protein degradation, due to the relatively slow conversion of resazurin to fluorescent resorufin (usually >1 h) or Gly-Phe-AFC (7-amino-4-trifluoromethylcoumarin) protease substrate (>30 min) within live cells. Generally, transcriptional assay requires 12–24 h for treatment of cells with test compounds, while small molecule-mediated protein degradation is detectable within several hours and in some cases, in less than one hour [4,[9], [10], [11], [12], [13]]. Thus, for our on-going research on small molecule-mediated protein degradation, we required a multiplex assay that monitors both cell viability (number) and luciferase assay within a short time, preferably after cell lysis to stop cellular processes, including degradation pathways, as this allows examination of degradation processes on a shorter time scale.

**Fig. 1:**
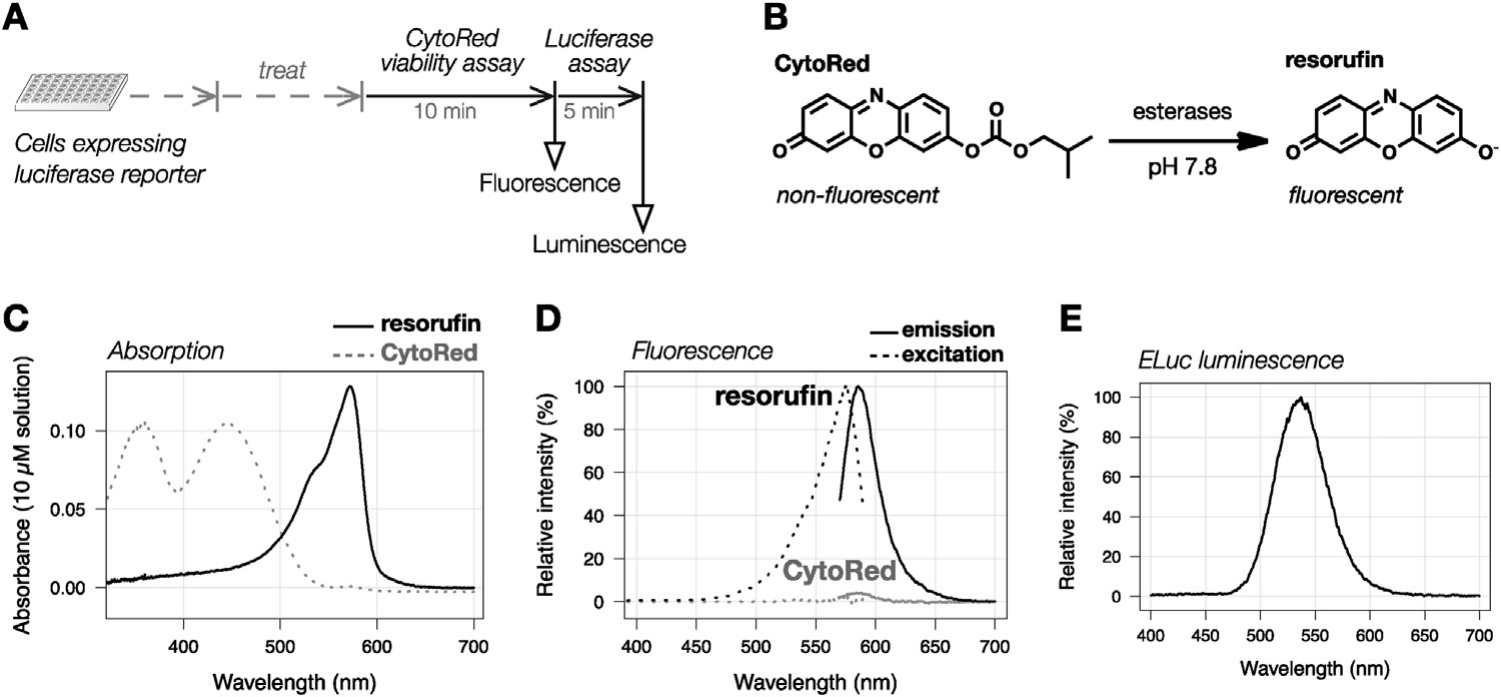
An overview of the developed method, and basic spectroscopic properties of CytoRed, resorufin and bioluminescence from Emerald Luc (ELuc). **(A)** Schematic illustration of the CytoRed-luciferase multiplex assay. Cells expressing a luciferase reporter were plated on multi-well plates, and CytoRed viability assay and luciferase assay were sequentially performed after desired treatment of the cells. CytoRed viability assay was performed by adding CytoRed working solution that contains 1% Triton X-100 and 20 μM CytoRed after removal of the medium. Conversion of CytoRed to resorufin by cellular endogenous esterases was monitored in terms of resorufin fluorescence for 10 min, and then luciferase activity was assayed by adding D-luciferin solution. **(B)** Conversion of CytoRed to resorufin. **(C)** Absorption spectra of CytoRed and resorufin in the assay buffer. **(D)** Excitation and emission spectra of CytoRed and resorufin in the assay buffer. CytoRed is almost non-fluorescent, but resorufin is highly fluorescent under the assay conditions. **(E)** Bioluminescence spectrum of the luciferase reaction catalyzed by ELuc under the assay conditions.

Here, we describe a multiplex luciferase assay protocol that allows sequential measurement of endogenous esterase activity as a measure of cell number and luciferase activity in cell lysate (Fig. 1A). Among available esterase substrates tested, we found that a fluorogenic esterase substrate CytoRed [14] was compatible with luciferase activity measurement (Fig. 1B–D). Basic characterization of the protocol was performed with cells stably expressing HaloTag protein fused with emerald luciferase (ELuc), a firefly-type luciferase derived from Brazilian click beetle (Fig. 1E) [[15], [16], [17]], and estrogen receptor alpha (ER) fused with ELuc (ER-ELuc) [4].

## Reagents and materials

• CytoRed (isobutyloxycarbonyloxy-3H-phenoxazin-3-one): Commercially available from Dojindo Molecular Technologies (as 1 mM DMSO solution, Cat. No. C410), Adipogen (Cat. No. CDX-I0005), or Sigma-Aldrich (Cat. No. 79972). Alternatively, this reagent can be synthesized from resorufin (Tokyo Chemical Industry, Cat. No. R0012) in one step as described previously (see supplementary methods) [14].
• D-Luciferin potassium salt, for molecular biology (FUJIFILM Wako Pure Chemicals, Cat. No. 12-05116)
• Coenzyme A trilithium salt, from yeast (FUJIFILM Wako, Cat. No. 305-50733)
• 3-Mercapto-1,2-propanediol, for molecular biology (FUJIFILM Wako Pure Chemicals, Cat. No. 139-16452)
• Adenosine 5′-triphosphate disodium salt, crystalline (FUJIFILM Wako Pure Chemicals, Cat. No. 303-50511)
• 0.5 M EDTA, pH 8.0 (FUJIFILM Wako Pure Chemicals, Cat. No. 311-90075)
• Tricine (Dojindo Molecular Technologies, Cat. No. GB19)
• Magnesium acetate tetrahydrate (FUJIFILM Wako Pure Chemicals, Cat. No. 130-00095)
• Triton X-100 (Nacalai Tesque, Cat. No. 35501-15)
• Dimethyl sulfoxide (DMSO), for biochemistry (FUJIFILM Wako Pure Chemicals, Cat. No. 046-21981)
• White 96-well plate with a clear bottom (Greiner, Cat. No. 655098)
• A plate reader capable of measuring luminescence and fluorescence (excitation 570 nm/emission 600 nm). The protocol described here employs a glow-type luciferase assay, and injectors are not required.
• Multichannel pipette (NICHIRYO, Nichipet Ex II MULTI 8-channel 5–100 μL, Cat. No. 00-NPM-8SP, or equivalent)
• Disposable reagent reservoir, 25 mL, divided, sterilized (VistaLab, Cat. No. 3054-1004)
• Aspirator (Biosan, Cat. No. FTA-2, or equivalent)
• Straight manifold for aspirator, 8-channel (Drummond Scientific, Cat. No. 3-00-093, or equivalent)

## Recipes

• 10 × TME buffer (300 mM Tricine-NaOH, 80 mM Mg(OAc)_2_, 2 mM EDTA, pH 7.8): Mix60 mL of 1 M Tricine-NaOH pH 7.8 (see recipe below), 16 mL of 1 M Mg(OAc)_2_ (see recipe below), and 0.80 mL of 0.5 M EDTA solution pH 8.0, and adjust volume to 200 mL with H_2_O to 200 mL. Filter-sterilize and store at 4 °C.
• 10% v/v Triton X-100: Dissolve 30 mL of Triton X-100 in 270 mL of H_2_O. Store at 4 °C.
• 10 mM D-luciferin stock solution: Dissolve 50 mg of potassium D-luciferin in 15.7 mL of H_2_O, store in small aliquots (e.g., 0.5 mL) at −20 °C. Minimize freeze & thaw cycles to several times.
• 100 mM ATP stock solution: Dissolve 551 mg of adenosine 5′-triphosphate disodium salt in 10 mL of H_2_O, and store in small aliquots (e.g., 0.5 mL) at −20 °C. Minimize freeze & thaw cycles to several times.
• 50 mM CoA stock solution: Dissolve 1.0 g of coenzyme A trilithium salt in 23.8 mL of H_2_O, and store in small aliquots (e.g., 0.5 mL) at −20 °C. Minimize freeze & thaw cycles to several times.
• 1 M Tricine-NaOH pH 7.8: Dissolve 35.8 g of tricine in 150 mL of H_2_O, and adjust the pH to 7.8 with 2 M NaOH (approximately 30 mL). Adjust the volume to 200 mL with H_2_O, filter-sterilize, and store at ambient temperature.
• 1 M Mg(OAc)_2_: Dissolve 21.5 g of magnesium acetate tetrahydrate in 100 mL of H_2_O, filter-sterilize, and store at 4 °C.
• 10 mM CytoRed stock solution: Dissolve 1.25 mg of CytoRed in 400 μL of DMSO, and store in small aliquots (e.g., 50 μL) at −20 °C. Minimize freeze & thaw cycles to several times, and protect from light.
• 20 μM CytoRed working solution (recipe for one 96-well plate): Mix0.6 mL of 10 × TME buffer, 0.6 mL of 10% Triton X-100, and 4.8 mL of H_2_O. Add 12 μL of 10 mM CytoRed stock solution, and vortex to make homogenous solution.
• Luciferin working solution (recipe for one 96-well plate): [1,4] Mix0.6 mL of 10 × TME buffer, 0.6 mL of 10% Triton X-100, and 4.45 mL of H_2_O. Add 50 μL of 1-mercapto-2,3-propandiol, 90 μL of 100 mM ATP stock solution, 60 μL of 50 mM CoA stock solution, and 150 μL of 10 mM D-luciferin stock solution. The solution contains 30 mM Tricine-NaOH, 8 mM Mg(OAc)_2_, 0.2 mM EDTA, 100 mM 3-mercapto-1,2-propandiol, 1% Triton X-100, 1.5 mM ATP, 0.5 mM CoA, and 0.25 mM D-luciferin. The working solution should be prepared just before use, and we recommend using the working solution within 30 min after preparation.

## Procedure for CytoRed-luciferase dual assay

The following is a representative procedure for a 96-well plate assay. Fig. 1A illustrates how this multiplex assay works.

1 Seed cell lines expressing a luciferase construct at a density appropriate for the luciferase assay, in a white 96-well clear-bottomed plate (100 μL/well), and incubate in a humidified CO_2_ incubator for an appropriate time.
2 If required, treat cells with test compounds for an appropriate time.
3 Prepare 20 μM CytoRed working solution and luciferin working solution as described in the recipes section. The working solutions are stable at least for an hour when protected from light.
4 Remove medium with an aspirator equipped with a multichannel adaptor. Proceed to the next step within a few minutes to avoid drying of the cells.
5 Add 50 μL/well of the CytoRed working solution to the plate with a multichannel pipette and a reagent reservoir, and immediately measure fluorescence on an appropriate microplate reader (Ex. 570 nm/Em. 615 nm, bottom read mode, EnVision plate reader from PerkinElmer).
6 Incubate the plate at room temperature or 37 °C for 10 min, and measure the fluorescence again. For analysis, subtract the initial fluorescence from the fluorescence after incubation. Alternatively, the plate reader’s kinetic measurement mode can also be used for automatic measurement. Note that longer incubation at this step results in weaker luminescence in the luciferase assay.
7 Add 50 μL/well of the luciferin working solution to the plate, and measure the luminescence after 1 min on an appropriate plate reader (we used an EnVision plate reader in ultra-sensitive luminescence mode with 10 msec exposure). Under the assay conditions (glow-type luciferase assay), luminescence lasts for more than 10 min, but gradually decreases in intensity.

## Method validation

**Fig. 2:**
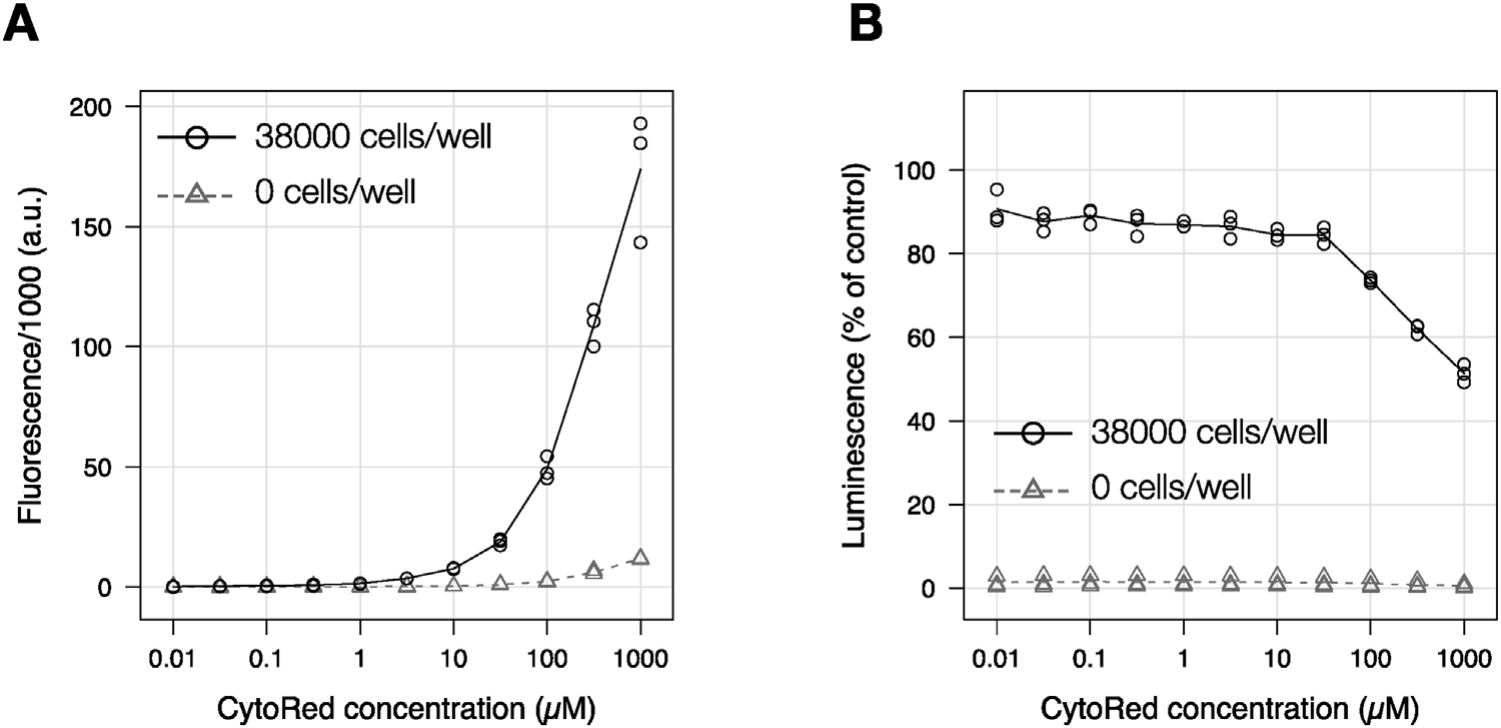
Optimization of CytoRed concentration in CytoRed-luciferase multiplex assay. HEK293 cells stably expressing HaloTag-ELuc under the control of cytomegalovirus (CMV) promoter were plated at 38,000 cells/well, which approximately corresponds to 40% confluency, and after adhesion of the cells to the plate (after 3 h) CytoRed-luciferase multiplex assay was performed with the indicated CytoRed concentrations. **(A)** Effect of CytoRed concentration on esterase activity measurement. The circles represent raw fluorescence from wells with the indicated number of cells, and the triangles represent fluorescence from wells without cells. The Z’-factor values at various CytoRed concentrations were as follows: −1.64 at 0.1 μM, −1.08 at 0.32 μM, 0.40 at 1 μM, 0.85 at 3.2 μM, 0.87 at 10 μM, 0.75 at 32 μM, 0.68 at 100 μM, 0.75 at 320 μM, and 0.50 at 1000 μM. **(B)** Effect of CytoRed concentration on luciferase assay performance. At less than 32 μM CytoRed, only negligible interference with the luciferase luminescence was observed.

First, we examined the impact of CytoRed concentration on the fluorescence intensity and the luminescence from luciferase. Cells stably expressing HaloTag-ELuc were first assayed for endogenous esterase activity with varying concentrations of CytoRed in a Triton X-100-based lysis buffer. After 10 min at room temperature, fluorescence was measured, and the luciferase activity was then assayed as described above. As shown in Fig. 2A, fluorescence from the hydrolyzed CytoRed steadily increased with increasing CytoRed concentration, and the fluorescence intensities from wells with or without cells were well separated at more than 3 μM CytoRed. The Z’ factor (0.5–0.87) indicated that the assay conditions were suitable for screening purposes [18]. CytoRed concentrations of less than 30 μM did not interfere with luciferase activity in luciferase assay of the same sample. Thus, we selected 20 μM CytoRed as an optimal condition for the multiplex assay. We also tested *p*-nitrophenyl acetate and *p*-nitrophenyl isobutyl carbonate as colorimetric esterase substrates, but a sufficient signal could not be achieved even with a longer incubation time (up to 1 h) or a higher concentration (1 mM) of the substrate, at which significant interference with the luciferase assay was observed. Note that a longer incubation period at this step diminishes luciferase activity even in the absence of CytoRed. So, we recommend keeping the incubation time as short as possible (10 min).

**Fig. 3:**
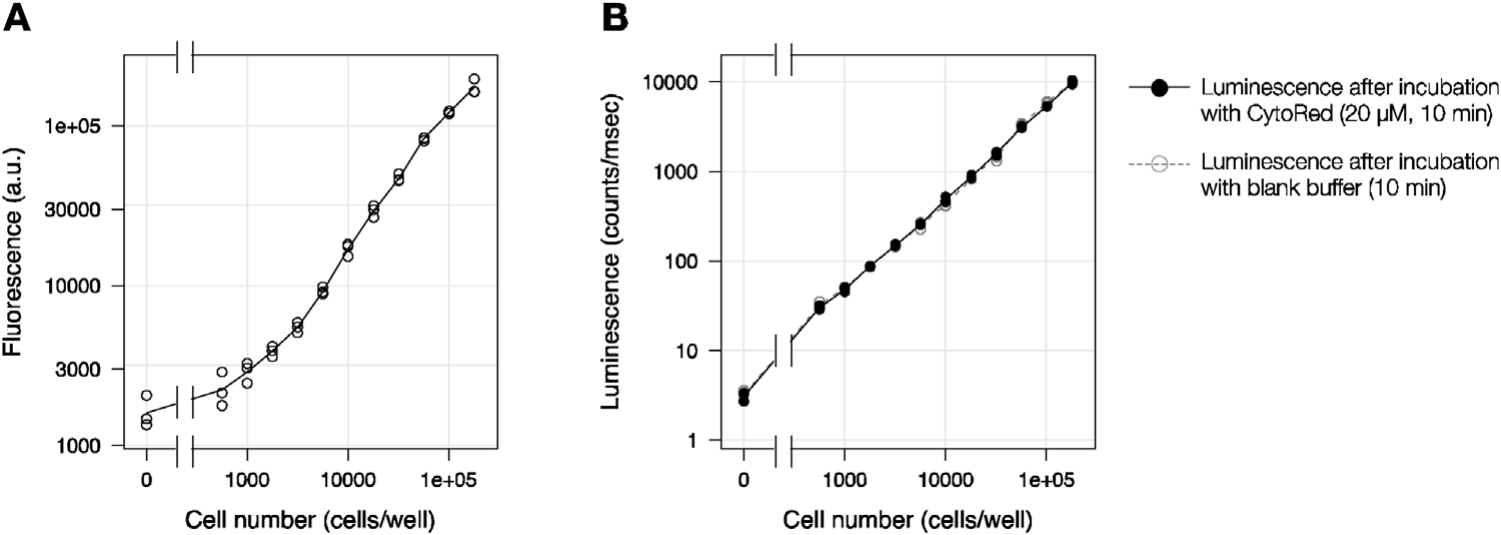
Dynamic ranges of CytoRed viability assay and luciferase assay under the multiplex assay conditions. Serially diluted HEK293 cells stably expressing HaloTag-ELuc were subjected to CytoRed-luciferase multiplex assay. **(A)** Dynamic range of CytoRed viability assay. Data points represent raw fluorescence intensity measured from triplicate wells. **(B)** Dynamic range of luciferase assay after CytoRed viability assay. Black filled circles represent luciferase luminescence from wells assayed with 20 μM CytoRed working solution, and gray open circles represent luciferase luminescence from wells assayed with CytoRed working solution without CytoRed.

Next, we examined the dynamic range of the CytoRed esterase assay and the subsequent luciferase assay, by using serially diluted cells expressing HaloTag-ELuc. As shown in Fig. 3A, CytoRed fluorescence increased with increasing cell density, and the increase was sufficiently linear for viability (cell number) assay within the range of 3000 cells/well to 100,000 cells/well, which corresponds to 3–100% confluency. The luciferase signal, which was measured after CytoRed assay, gave excellent linearity over more than 2 orders of magnitude of cell density (Fig. 3B). Additionally, no difference in luminescence intensity was observed in the presence or absence of CytoRed, thus supporting the compatibility of CytoRed assay with luciferase assay.

**Fig. 4:**
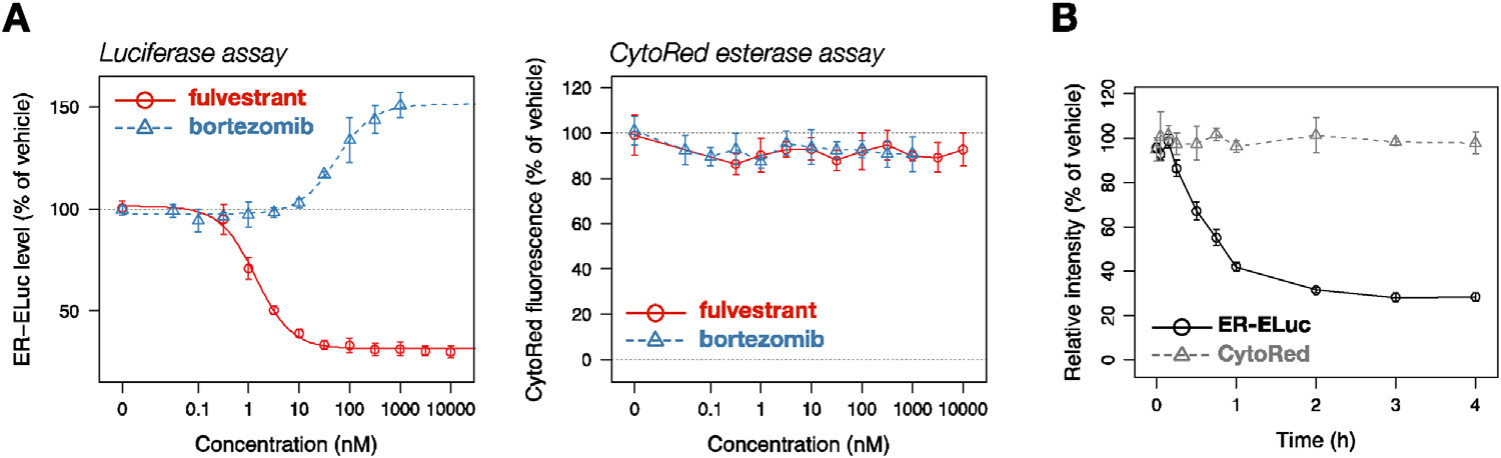
Applications of CytoRed-luciferase multiplex assay for dose-response analysis and kinetic analysis of ER degradation. HEK293 cells stably expressing ER-ELuc under the control of CMV promoter [4] were plated on a 96-well plate and cultured until 70% confluency. The cells were then treated with either fulvestrant or bortezomib for 4 h at the indicated concentration (A), or treated with 10 μM fulvestrant for the indicated time (B). Cell viability (relative cell number) and luciferase activity were evaluated with the CytoRed-luciferase multiplex assay. **(A)** Dose-dependent modulation of ER-ELuc level by fulvestrant and bortezomib. The left panel shows the results of luciferase assay and the right panel shows the results of CytoRed assay. Red circles represent mean values from fulvestrant-treated cells and blue triangles represent mean values from bortezomib-treated cells. Error bars denote standard deviation (n = 3). **(B)** Kinetics of fulvestrant-induced degradation of ER-ELuc. Black circles denote mean luminescence intensity from ER-ELuc and the error bars denote standard deviation (n = 3). Gray triangles denote mean cell viability determined by CytoRed assay, and the error bars denote standard deviation (n = 3).

Finally, we demonstrate the utility of this protocol by showing its suitability for dose-response analysis and kinetic analysis of ER degradation induced by fulvestrant, a representative selective estrogen receptor degrader (SERD) [9]. As shown in Fig. 4A, the down-regulatory effect of fulvestrant on ER-ELuc could be reproduced with this protocol, which gave an IC_50_ value of 1.4 nM, in good agreement with our previously determined value (1.4 nM) [4]. Also, bortezomib, a proteasome inhibitor, increased the ER-ELuc level in a dose-dependent manner with its EC_50_ value being 55 nM, consistent with the proteasomal degradation of ER proteins. Furthermore, the kinetics of the degradation could be examined with this endpoint assay (Fig. 4B), and the half-life of the ER-ELuc in the presence of 10 μM fulvestrant was estimated to be around 30 min. Notably, no degradation was detected at less than 10 min treatment, implying the presence of a short induction period or delay that may reflect the time required for processes such as distribution of the compound into cells, binding to the receptor, and steps required for proteasomal degradation.

In summary, we have developed and validated a multiplex assay that sequentially monitors endogenous esterase activity and luciferase activity. This endpoint multiplex assay protocol takes only an additional 15 min compared to conventional luciferase assay, and allows monitoring of fast processes, providing an alternative to luciferase assay in live cells [7], which results in a significantly weaker luminescence signal. Thus, the CytoRed-luciferase multiplex assay described here is expected to be especially useful for researchers working on stable cell lines expressing luciferase constructs.

## Additional information

The indicated order of measurement of esterase activity with CytoRed fluorescence and then luciferase activity is essential for success. Our attempts to measure esterase activity after luciferase activity measurement failed, possibly due to inactivation of esterase during luciferase activity measurement.
