# Supplementary material for: Multiplexing fluorogenic esterase-based viability assay with luciferase assays
Source: MethodsX. 2019 Sep 12;6:2013–20. doi: 10.1016/j.mex.2019.09.008 (PMC6812399; doi:10.1016/j.mex.2019.09.008)
Supplement: Supplementary file 1 [file mmc1.docx]

**Supplementary methods**

General methods for organic synthesis:

Nuclear magnetic resonance (NMR) spectra were recorded on a JEOL JNM-ECA-500 (500 MHz) spectrometer. Chemical shift values were expressed in δ values (ppm) relative to internal tetramethylsilane (0.00 ppm) or residual CHCl_3_ (7.24 ppm) for ^1^H NMR and internal tetramethylsilane (0.00 ppm) or residual CHCl_3_ (77.0 ppm) for ^13^C NMR. Thin-layer chromatography was performed on silica gel 60 F254 plates (Merck) and bands were visualized with UV (254 nm), iodine vapor, and phosphomolybdic acid stain.

Synthesis of CytoRed (7-Isobutyloxycarbonyloxy-3*H*-phenoxazin-3-one):

The title compound was prepared as described previously [14]. A solution of resorufin (194 mg, 0.910 mmol) and 4-dimethylaminopyridine (4.9 mg, 0.040 mmol, 4 mol%) in dehydrated THF (9 mL) was sequentially treated with triethylamine (0.38 mL, 2.7 mmol) and isobutyl chloroformate (0.60 mL, 4.6 mmol), and heated to 55 ºC for 3 h. The reaction was quenched by adding water and saturated aqueous sodium bicarbonate, and extracted with ethyl acetate. The organic layer was separated, washed (saturated aqueous sodium bicarbonate and brine), and dried over magnesium sulfate. The solvent was evaporated, and the dark red residue was purified by flash silica gel chromatography (dichloromethane / ethyl acetate 10:1) to afford an orange powder. The powder was recrystallized from a mixture of dichloromethane and hexane to afford CytoRed (74.4 mg, 0.237 mmol, 26% yield) as an orange powder. TLC (dichloromethane / ethyl acetate 5:1): R_f_ = 0.4; ^1^H-NMR (500 MHz, CDCl_3_) δ: 7.81 (d, *J* = 8.6 Hz, 1H), 7.44 (d, *J* = 9.7 Hz, 1H), 7.26 (d, *J* = 2.9 Hz), 7.22 (dd, *J* = 8.9, 2.6 Hz, 1H), 6.87 (dd, *J* = 9.7, 1.7 Hz, 1H), 6.34 (d, *J* = 1.7 Hz, 1H), 4.09 (d, *J* = 6.9 Hz, 2H), 2.12–2.04 (m, 1H), 1.03 (d, *J* = 6.9 Hz, 6H); ^13^C-NMR (125 MHz, CDCl_3_) δ: 186.27, 153.64, 152.70, 149.26, 148.38, 144.34, 135.20, 134.78, 131.23, 131.20, 118.61, 109.11, 107.28, 75.40, 27.75, 18.84 (2C).
